# Risk interaction of obesity, insulin resistance and hormone-sensitive lipase promoter polymorphisms (LIPE-60 C > G) in the development of fatty liver

**DOI:** 10.1186/1471-2350-14-54

**Published:** 2013-05-20

**Authors:** Pi-Jung Hsiao, Zhih-Cherg Chen, Wei-Wen Hung, Yi-Hsin Connie Yang, Mei-Yueh Lee, Jee-Fu Huang, Kung-Kai Kuo

**Affiliations:** 1 Department of Internal Medicine, Division of Endocrinology and Metabolism, Kaohsiung Medical University Hospital, Kaohsiung, Taiwan; 2 Department of Medical Research, Section of Internal Medicine, Chi-Mei Medical Center, Tainan, Taiwan; 3 Biostatistics, in the Statistical Analysis Laboratory, Kaohsiung Medical University Hospital, School of Pharmacy, Kaohsiung, Taiwan; 4 Department of Internal Medicine, Hepatobiliary Division, Kaohsiung Medical University Hospital, Kaohsiung, Taiwan; 5 Department of Surgery, Hepatobiliary Division, Kaohsiung Medical University Hospital, Kaohsiung, Taiwan; 6 School of Medicine, College of Medicine, Kaohsiung Medical University, Kaohsiung, Taiwan; 7 Department of Surgery, Division of Hepato-Bilio-Pancreatic Surgery, Kaohsiung Medical University, Hospital 100 Tzyou 1st Rd, Kaohsiung 807, Taiwan

**Keywords:** Non-alcoholic fatty liver disease (NAFLD), Hormone-sensitive lipase (HSL), Insulin resistance, Metabolic syndrome

## Abstract

**Background:**

Hormone sensitive lipase (HSL) promoter (LIPE-60 C > G) polymorphism has been found to be involved in hepatic steatosis, obesity, diabetes and dyslipidemia. The precise interactions between these risk factors and genetic susceptibility that may affect non-alcoholic fatty liver disease (NAFLD) are still not fully determined.

**Methods:**

A cross-sectional study was conducted in 1056 men. To avoid the confounding effect of plasma glucose, the study population was classified into normal glucose tolerance (NGT, n = 729) and glucose intolerance (GI, n = 299) groups. NAFLD was diagnosed by abdominal ultrasound after ruling out any history of alcohol abuse. A multivariate regression model was used to estimate the impact of these factors on NAFLD.

**Results:**

In the NGT group, subjects with NAFLD often have complicated metabolic abnormalities. The coexistence of NAFLD and GI has been demonstrated to have a synergistic effect raising BMI, serum insulin and HOMA-insulin resistance (HOMA-IR). BMI and adipose-insulin resistance (Adipo-IR), but not HOMA-IR, significantly contributed to a greater risk of developing NAFLD. Serum triglyceride was significantly up-regulated in men with the (CG + GG) genotype of HSL promoter polymorphism, NAFLD and Adiopo-IR in sequence.

**Conclusion:**

Adipo-IR, rather than HOMA-IR, appears to be a consistent insulin resistance index in the study of NAFLD. G allele of the HSL promoter polymorphism may contribute the greatest impact raising serum triglyceride in a state of glucose intolerance.

## Background

Non-alcoholic fatty liver disease (NAFLD) is the most prevalent chronic liver disease in the world, and is growing especially quickly in Asia. It is regarded as a hepatic manifestation of metabolic syndrome (MetS) because of its close concomitance with obesity, type 2 diabetes mellitus and dyslipidemia [1,2]. Patients with NAFLD, who are at increased risk of atherosclerotic cardiovascular disease, are also at risk of progression to non-alcoholic steatotic hepatitis (NASH), cirrhosis or hepatocellular carcinoma [3,4]. Our previous study clearly documented a very high prevalence (57.8%) of fatty liver, as assessed by abdominal ultrasound, in adult Taiwanese [5]. Previous study has also shown that compared to Caucasians, Asians (mostly Chinese) have a greater fat accumulation in the body and viscera for a given body mass index (BMI) [6]. Understanding the consequences and how to prevent the development of fatty liver are extremely important for Asians.

NAFLD, defined as hepatic steatosis with an intrahepatic triglyceride (TG) content > 5% of the liver volume or weight, develops owing to an imbalance between fatty acid (FA) input and output. Physiologically, the hepatic TG content results from a complex interaction of lipid homeostasis, including (1) fatty acid influx derived by adipose lipolysis (contributing 60% of hepatic FA), (2) dietary fat intake from chylomicron (10- 15%), (3) *de novo* lipogenesis from plasma glucose (25- 30%), (4) fatty acid β-oxidation and (5) fatty acid export by esterification to secrete as a very low-density lipoprotein (VLDL) [7,8]. The mechanism of excess hepatic fat accumulation is attributed generally to enhanced FA delivery from adipose lipolysis and increased *de novo* lipogenesis in the liver itself, while β-oxidation and VLDL export play minor roles [8].

Fatty acid synthase (FAS), catalyzing the final step in FA biosynthesis, is well known to be the major determinant of the generation of hepatic FA by *de novo* lipogenesis. Altered FAS expression has been correlated with obesity related insulin resistance and hepatic steatosis. Therefore, circulating FAS has been suggested to be a possible surrogate marker of insulin resistance [9,10]. In the FA metabolism, adipose triglyceride lipase (ATGL) and hormone-sensitive lipase (HSL) are responsible for > 95% of TG hydrolysis. Both ATGL and HSL regulate the basal lipolysis, whereas only HSL determines the stimulated lipolysis. HSL, catalyzing diacylglycerol and monoacylglycerol into free fatty acids, determines the rate-limiting step to modulate complete lipolysis [11,12]. HSL is also engaged in the mobilization of FA from intracellular lipid stores in tissues. Insulin represents the most potent inhibitor of HSL to shut down lipolysis, and HSL expression has often been correlated with the pathogenesis of type 2 diabetes, abdominal obesity and MetS [13-15]. Insulin resistance is the pathophysiologic hallmark of the development of NAFLD. As there is a very low expression of ATGL in the liver, the activities of FAS and HSL seem to be essential for the regulation of fatty acid metabolism in the formation of NAFLD.

Genetic susceptibility to hepatic lipid accumulation is also considered important because of the evidence that approximately one-third of NAFLD occurs in subjects without the documented risk factors of obesity and insulin resistance. The *Ile* 1483 variant of the FAS gene was reported to have a protective effect, with a lower BMI, waist hip ratio, fasting glucose and blood pressure [16]. The well-studied promoter variant of HSL (−60 C > G), exhibiting a 40% decline in promoter activity, plays a critical role in fat metabolism in some diseases in a sex-, race- and insulin-dependent manner [17,18].

A combination of genetic and environmental risk factors, for example, diet, obesity or diabetes, is well known to cause the development of NAFLD [4,19]. However, the risk interaction and the relative impact on the development of NAFLD of individual genes and related metabolic biomarkers have not been thoroughly investigated. We designed this study to clarify the impact of metabolic abnormalities on the relationship between fatty liver and glucose intolerance. The differential impact of confounding risks for the development of NAFLD was analyzed after stratification of the fasting glucose. The results could have eventual clinical utility to help establish a practical treatment strategy for NAFLD in distinct populations with normal or abnormal glucose tolerance.

## Methods

### Selection criteria

Subjects were recruited from the Department of Preventive Medicine at KMUH in 2005 under the approval and supervision of the Institutional Review Board of Kaohsiung Medical University Hospital (KMUH-IRB-980323). All of the serum was obtained from the tissue bank in our hospital and de-identified from participant’s names and personal characteristics. To avoid gender bias, a cross-sectional population of 1056 males was randomly enrolled within 3 months. The detailed medical history of each subject was evaluated by an experienced physician. Twenty-seven participants were excluded due to recognized dyslipidemia secondary to poorly-controlled DM, documented DM with medication, Cushing’s syndrome, hypothyroidism, nephrotic syndrome, chronic liver disease, heavy alcohol use (drinking more than once weekly) or use of lipid-lowering agents. A total of 1029 male subjects were eligible for further study, and were stratified by fasting glucose into normal glucose tolerance (NGT, < 100 mg/dl) and glucose intolerance (GI, ≥ 100 mg/dl) groups.

### Laboratory measurements

After overnight fasting, blood samples were collected and analyzed for serum glucose, aspartate aminotransferase (AST), alanine aminotransferase (ALT), total cholesterol, serum triglyceride, HDL-cholesterol, and LDL-cholesterol, using a multichannel autoanalyser. Serum insulin was measured using commercial radioimmunoassay kits (Diagnostic Products Corporation, Los Angeles, CA, USA). Serum non-esterified fatty acid (NEFA) was measured by colorimetry (Wako Pure Chemical Industries, Ltd, Osaka, Japan). The objectively quantitative expression of the relative hepatic insulin resistance was indicated by the homeostatic model assessment of insulin resistance (HOMA-IR index = insulin (μU/mL) × glucose (mmol/L)/22.5). The adipose insulin resistance was expressed as the adipose insulin resistance (Adipo-IR index = fasting serum NEFA (μmol/L) × fasting serum insulin [μU/mL]) [20,21].

### Search strategy

EDTA-treated blood samples were used for DNA extraction by standard methods. The TaqMan genotyping assay was performed to detect the sequence of fatty acid synthase *FAS* polymorphisms (**rs2228305** for Ile1483Val, **rs2228307** for Val1888Ile) and *HSL* promoter polymorphism (**rs34845087** for C-60G, *LIPE* 14672 C > G). These assays were designed according to the SNP reference data in the NCBI GenBank database. The ABI PRISM 7500® sequence detection system was use to determine the sequence of the gene variants (Applied Biosystems, Roche, Taipei, Taiwan).

### Evaluation of fatty liver

Sonographic diagnosis of fatty liver was performed by abdominal B-mode ultrasound (3.5 MHz convex transducer, Toshiba SSA-250; Toshiba, Tokyo, Japan) carried out by experienced hepatologists trained at the same institution to ensure interobserver consistency. Diagnosis of fatty liver was based on the brightness of the liver on ultrasound as compared with the kidney, vascular blurring of the hepatic vein trunk, and deep attenuation in the right hepatic lobe. The absence of fatty liver change (AFL) was defined as a normal echo texture without visible fatty change. The presence of fatty liver (FL) was defined as an increase in the fine echoes of hepatic parenchyma with impaired visualization of the intrahepatic vessels and diaphragm.

### Statistical analysis

The SPSS 18.0 statistical package for Windows (SPSS Inc., Chicago, IL, USA) was used for all of the statistical analyses. Continuous variables were represented as the means ± SD. Nonparametric tests (Kruskal–Wallis test or Mann–Whitney rank-sum test) were used when the original measurements were highly skewed. Allele frequency was estimated by direct counting, while genotype distribution with Hardy–Weinberg equilibrium was tested using the chi-square test. Two-way analysis of variance (ANOVA, α = 0.05) was carried out to evaluate the metabolic profiles by the interaction effects between fatty liver and glucose intolerance. Student’s *t*-test with Bonferroni comparisons post hoc analysis was conducted within the NGT and GI groups. Multivariate regression analysis was further employed using fatty liver as a dependent variable, while body mass index (BMI), HOMA-IR, Adipo-IR and HSL (GG + GC vs CC) genotype were selected as independent variables based on significance in univariate analyses. To avoid multicollinearity in the regression model, serum insulin and NEFA were not included as independent variables in the multivariate regression model. Separate multiple regression analyses stratified by fasting glucose were further used to evaluate the effects of BMI, HOMA-IR, Adipo-IR, fatty liver, and HSL promoter genotypes (GG + GC vs CC) on serum TG. In addition, to compare the parameter estimates between NGT and GI, a single multiple regression model was conducted with the additional interactions of glucose intolerance vs BMI, HOMA-IR, Adipo-IR, fatty liver, and HSL promoter (GG + GC vs CC). Statistical significance was defined as a *P* value of < 0.05 using a two-tailed test.

## Results

To standardize the *de novo* lipogenesis by fasting plasma glucose, our purely male population was divided into NTG (n = 729) and GI (n = 299) groups. The age of the participants ranged from 20 to 70 years, the majority (>80%) being distributed in the range of 40–65 years. The prevalence of GI was 29.1% in our adult population. There was a high prevalence of MetS abnormalities (obesity 30.6%, GI 29.1%, hyperTG 42.2%, low HDL-C 19.6% and hypertension 51.6%) in subjects with NAFLD. Minor allele A of FAS (Val1483Ile) and G of FAS (Val1888Ile) polymorphism was nearly absent, with a monogenic distribution of Val1483 (99.6%) and Val 1888 (100%). The genetic effect of FAS was not further analyzed in the development of fatty liver. The frequency of the minor G allele of the HSL promoter (−60 C > G) was 9.9%, while the genotype frequency of CC: CG: GG was distributed as 80.8: 18.4 : 0.8% in Hardy–Weinberg equilibrium. There was no significant difference in the frequency distribution of the HSL promoter (−60 C > G) genotype between the NGT and GI groups.

As shown in Table 1, the prevalence of FL (67.2%) in the GI group was significantly higher than in the NGT group (55.7%) (*p* = 0.001, chi-square test). Within the NGT or GI groups, there were significantly greater metabolic abnormalities in the presence of FL. The metabolic profiles, such as BMI, serum insulin and HOMA-IR, were significantly attributed to a synergistic effect of FL and GI. However, the metabolic abnormalities in the group of NGT and FL seemed equivalent or even worse than those in the GI group without FL. The metabolic abnormalities occurred more in the presence of FL.

**Table 1:** Comparison of the metabolic abnormalities between glucose tolerance and fatty liver

**Groups/variables**	**Normal Glucose Tolerance (NGT)**		**Glucose Intolerance (GI)**		***P *for effect of FL vs AFL**	***P *for effect of GI vs NGT**	***P *for interaction of FL and GI**
	**AFL (n = 323)**	**FL (n = 406)**	**AFL (n = 98)**	**FL (n = 201)**			
Age (yrs)	45.63 ± 8.10	44.60 ± 8.65	48.68 ± 6.89	49.21 ± 8.69	0.675	< 0.0001	0.195
BMI (kg/m^2^)	23.16 ± 2.51	25.64 ± 3.06*	22.79 ± 3.31	26.16 ± 3.03*	< 0.0001	0.716	0.035
Serum insulin (μIU/ml)	2.22 ± 2.47	4.28 ± 4.48*	3.77 ± 6.73	7.74 ± 12.00*	< 0.0001	< 0.0001	0.044
NEFA (μmol/L)	509.81 ±192.78	575.03 ± 227.93*	589.47 ±230.07	646.75 ± 264.183	< 0.0001	< 0.0001	0.811
HOMA-IR	0.48 ± 0.53	0.94 ± 0.99*	1.28 ± 2.89	2.47 ± 4.49*	< 0.0001	< 0.0001	0.027
Adipo-IR	1.11 ± 1.22	2.57 ± 3.95*	2.10 ± 3.86	4.50 ± 5.24*	< 0.0001	< 0.0001	0.083
SBP (mmHg)	123.5 ± 15.3	126.2 ±15.1*	126.86 ± 16.86	130.44 ±18.20	0.007	0.001	0.701
DBP (mmHg)	76.2 ± 10.9	79.1 ± 10.6*	78.55 ± 11.49	82.45 ± 11.83*	< 0.0001	< 0.0001	0.530
AST (IU/L)	27.20 ±11.81	29.97 ± 11.41*	28.77 ±16.31	34.22 ± 18.71*	< 0.0001	0.004	0.163
ALT (IU/L)	25.28 ± 19.78	34.35 ± 20.64*	25.94 ± 20.91	40.70 ± 33.01*	< 0.0001	0.037	0.090
Cholesterol (mg/dl)	175.13 ± 31.00	184.24 ± 32.65*	173.98 ± 28.97	188.17 ± 34.57*	< 0.0001	0.548	0.274
Triglyceride (mg/dl)	104.78 ± 58.24	158.29 ± 115.18*	129.99 ± 111.28	163.97 ± 102.82*	< 0.0001	0.028	0.165
HDL-C (mg/dl)	53.75 ± 12.87	49.12 ± 11.55*	50.09 ± 10.67	48.30 ± 10.68	0.002	0.034	0.176
LDL-C (mg/dl)	115.27 ± 31.50	130.27 ± 32.97*	117.23 ± 28.96	129.08 ± 33.12*	< 0.0001	0.895	0.584

*P < 0.025 = 0.05/2 (Bonferroni test, two sample comparison within the normal glucose tolerance and glucose intolerance groups).

AFL indicates absence of fatty liver, FL indicates fatty liver. BMI indicates body mass index. SBP and DBP indicate systolic and diastolic blood pressure.

NEFA indicates non-esterified fatty acid.

In the development of FL, risk analysis was conducted to compare the odds ratios of BMI, HOMA-IR, Adipo-IR and HSL promoter genotypes (CC vs CG + GG) (Table 2). Analysis showed that BMI and Adipo-IR, rather than HOMA-IR and HSL promoter polymorphism, are independent risk factors for the formation of FL.

**Table 2:** Logistic regression using fatty liver as a dependent variable in normal glucose tolerance and glucose intolerance groups

**Independent variables**	**Odds ratio**	***P *value**
**Normal glucose tolerance**		
BMI	1.31 (1.22-1.41)	< 0.0001
HOMA-IR	0.98 (0.55-1.77)	0.948
Adipo-IR	1.36 (1.06-1.75)	0.016
HSL promoter genotype (CC vs CG + GG)	1.41 (0.71-2.16)	0.122
**Glucose intolerance**		
BMI	1.50 (1.32-1.69)	< 0.0001
HOMA-IR	0.85 (0.64-1.13)	0.258
Adipo-IR	1.41 (1.11-1.78)	0.005
HSL promoter genotype (CC vs CG + GG)	1.03 (0.49-2.18)	0.940

Obesity plays a central role in MetS. Our study demonstrated that the frequency of FL and the metabolic profiles of MetS were positively parallel to BMI, with the exception of GI (Table 3). The frequency of FL is greater than that of GI for a given BMI. Relevant metabolic abnormalities, including 38.4% for fatty liver, 33.4% for hypertension, 26.4% for glucose intolerance, 18.2% for hypertriglyceridemia and 10.1% for low HDL-C, existed in normal BMI subjects; this has previously been regarded as metabolic obese normal weight (MONW). This means that hepatic steatosis is not only dependent on the BMI status, and the threshold for hepatic fat accumulation essentially differs greatly between individuals.

**Table 3:** Prevalence of fatty liver and indexes of metabolic syndrome by BMI status

**BMI status (kg/m^2^)**	**< 24 (n = 434)**	**24- 26.9 (n = 379)**	**≥ 27 (n = 216)**	***P***
Fatty liver	38.4%	67.3%	86.1%	< 0.0001
Hypertension	33.4%	53.0%	57.9%	< 0.0001
Glucose intolerance	26.4%	29.2%	34.9%	0.086
Hypertriglyceridemia	18.2%	37.4%	49.3%	< 0.0001
HDL < 40 mg/dl	10.1%	22.6%	24.6%	< 0.0001

Hypertension is defined as systolic BP ≥ 130 or diastolic BP ≥ 80 mmHg.

Glucose intolerance is defined as fasting glucose ≥ 100 mg/dl.

Hypertriglyceridemia is defined as fasting triglyceride ≥ 150 mg/dl.

Power calculation was more than 80% in the whole population when the pooled standard deviation of TG is estimated around 90 mg/dl. In the NGT group, the presence of FL followed by high Adipo-IR and BMI significantly contributed to a raised serum TG (Table 4). However, the effect of BMI on serum TG disappeared in the GI group. Conversely, the HSL promoter genotype (CG + GG vs CC) demonstrated a significant strong impact elevating serum TG in the GI but not the NGT group. Comparison of the parameter estimates of the impact on serum TG, indicated by interactions, showed a significant difference in the HSL promoter (GG + GC vs CC) (*p* = 0.005) between the NGT and the GI groups. Therefore, it means that the TG level is very dependent upon the status of the subject’s glucose in-tolerance or not.

**Table 4:** Multiple regression using triglyceride as a dependent variable in the normal glucose tolerance and glucose intolerance groups

**Independent variables**	**Parameter estimates (B)**	**95% Confidence interval**		***P *value**
		**Lower bound**	**Upper bound**	
**Normal glucose tolerance**				
BMI	4.30	2.41	6.20	< 0.0001
HOMA-IR	−31.06	−41.11	−21.01	< 0.0001
Adipo-IR	17.04	14.35	19.74	< 0.0001
FL vs AFL	27.50	16.41	38.58	< 0.0001
HSL promoter genotype (CG + GG vs CC)	−3.05	−16.14	10.04	0.648
**Glucose intolerance**				
BMI	−0.32	−3.82	3.82	0.860
HOMA-IR	−11.01	−16.26	−5.76	< 0.0001
Adipo-IR	11.50	7.04	15.96	< 0.0001
FL vs AFL	29.40	3.74	55.05	0.025
HSL promoter genotype (CG + GG vs CC)	34.10	9.16	60.04	0.010

FL indicates fatty liver, AFL indicates absence of fatty liver.

## Discussion

Our study demonstrated that BMI and Adipo-IR significantly contributed to FL formation. Men’s BMI is reported to predict approximately 93% of the variance linked to obesity-related adipocyte size, fat cell mass and fat distribution [22]. Accordingly, BMI could be used as a clinically practical parameter in males to represent the obesity-related fat metabolism. There is substantial evidence that obesity is related to excess fat cell mass leading to increased basal lipolysis [11]. Insulin resistance is well-documented as the hallmark of MetS, but occurs with a differential response intensity of target tissues such as adipose, liver and muscle tissues to a given insulin level [23]. The Adipo-IR index is a simple way of measuring the inhibiting response of lipolysis by insulin in adipose tissue. Our results rationally suggest that people with a higher BMI usually have a greater fat cell mass, increased basal lipolysis leading to higher NEFA and greater insulin resistance (represented by Adipo-IR), which will result in hepatic FFA overflow and FL formation. The genetic effect of the HSL promoter (CC vs CG + GG) in relevant lipolysis and FL formation may be overcome by the greater impact of obesity-related lipolysis and insulin resistance of adipose (Adipo-IR). This finding is compatible with the fact that increased plasma FFA from adipose lipolysis is the major provider of lipids in the steatotic liver [8,20,24].

NAFLD has been regarded as the hepatic manifestation of MetS. However, debate continues regarding the progression of MetS to NAFLD or vice versa [25,26]. NAFLD has been reported to be a better independent risk factor for cardiovascular disease than MetS in some studies [3,27]. Liver fat is an independent determinant of the hepatic glucose output, which is sensitive to insulin. The liver is the primary site regulating 50-70% of insulin clearance [28]. Increased hepatic steatosis is associated with impaired insulin clearance and hepatic insulin resistance, which results in plasma glucose elevation, compensatory hyperinsulinemia and progression to type 2 DM. Hepatic insulin resistance also accelerates VLDL secretion, contributing to hypertriglyceridemia and low-HDL cholesterol concentration. Thus, hyperinsulinemia is regarded to be a consequence rather than a cause of NAFLD. Furthermore, hyperinsulinemia, stimulating the sympathetic tone and enhancing renal sodium reabsorption, contributes to hypertension [28,29]. HOMA-IR, an indirect estimate of insulin resistance, is usually used to represent the inhibition of hepatic glucose output by insulin, which is maintained by a balance between the liver and β-cells [20]. As suggested by Kotronen et al., the HOMA-IR index, which overestimates the insulin resistance in subjects with NAFLD, is more likely to reflect impaired insulin clearance and secretion than insulin sensitivity [29]. Our results revealed an inconsistent effect of HOMA-IR, whereas Adipo-IR showed a consistent trend in regulating NAFLD formation or serum TG. This highly suggests Adipo-IR as an index of insulin sensitivity in NAFLD or MetS research. In our study, subjects with FL and NGT complicated more metabolic abnormalities than subjects with AFL and GI. The coexistence of FL and GI exerts a significant synergistic effect raising BMI, serum insulin and HOMA-IR. This indicates that FL may develop earlier in the progression of MetS and the coexistence of FL and GI may magnify obesity and insulin resistance.

No matter how MetS is defined, visceral obesity is an obligatory component of MetS based on the recommendations of the International Diabetes Federation consensus [30]. However, there is still no convenient, practical worldwide indicator to define visceral obesity. As our study has shown, metabolic abnormalities are considerably prevalent in a MONW population. Visceral obesity is not easily defined by BMI alone since the threshold for hepatic lipid accumulation (a critical visceral adiposity) differs individually [4]. The liver is a pivotal organ to regulate the homeostasis of fat and glucose. It also plays a central role linking the interaction of obesity, GI, hypertension and dyslipidemia. Previous evidence has shown that hepatic steatosis rather than extrahepatic adiposity is more correlated with insulin resistance, especially in normal-weight non-diabetic subjects. NAFLD is an early manifestation of MetS and its severity is positively parallel to the degree of obesity. Therefore, hepatic steatosis may be the earliest sign in the pathogenesis of MetS and may be a better marker of visceral obesity for defining MetS, especially in a MONW population [4,26,31]. Compared with the gold standard of liver biopsy to diagnose FL, abdominal ultrasound is a noninvasive, convenient and accurate tool with high sensitivity and specificity. Therefore, we propose that a steatotic liver evaluated by ultrasound is a more sensitive indicator than BMI for defining visceral obesity.

Facing an increased FA influx and *de novo* lipogenesis, the hepatic FA pool is regulated by β-oxidation, with biosynthesis of TG for secretion as VLDL-C particles or storage as intrahepatic lipid. Current evidence suggests that hepatic TG synthesis and VLDL-TG secretion protect against lipotoxicity by buffering hepatic FFA influx [8]. Fasting serum TG is carried predominantly in the particles of VLDL secreted from the liver, which is inhibited by insulin. In subjects without FL, nearly 70% of FA incorporated into VLDL-TG is derived from plasma FA sources, and the rest originates from hepatic *de novo* lipogenesis and lipolysis of intrahepatic lipids. The VLDL-TG secretion rate is greater in subjects with FL than those without FL [4,7]. Our results demonstrated that the impact of increased circulating TG is significantly regulated by the presence of FL, Adipo-IR and BMI in sequence. This is compatible with the reported fact that a higher BMI, greater insulin resistance to adipose (Adipo-IR) and more liver fat is compensated with higher secretion of VLDL-TG [4,7,8,30]. Therefore, the presence of FL essentially could result in dyslipidemia and related atherosclerosis. Our results demonstrated a differential intensity of HOMA-IR inhibition of VLDL-TG secretion in the NGT and GI groups. In the GI state, it still demonstrated an inhibiting impact on VLDL-TG secretion coexistent with the impaired hepatic output in a given HOMA-IR, which implies differential insulin sensitivity to regulate fat and glucose metabolism in the liver, such as by inhibiting VLDL-TG secretion and hepatic glucose output. However, greater insulin resistance has been shown to lead to greater VLDL-TG secretion and higher serum TG [12-14]. Thus our variable TG regulation responses when using HOMA-IR as an insulin resistance index suggest the need for a more appropriate index to represent insulin resistance for glucose or fatty acid metabolism. Adipo-IR, representing the circulating FFA influx relative to insulin, can be regarded as a good indicator of insulin resistance in studies of TG metabolism and NAFLD.

There are many reports in the literature investigating C-60G gene polymorphism in the HSL promoter. The Ely study showed a gender specific effect on insulin and lipid levels in–60G carriers. Men carrying the –60G allele had significantly lower fasting NEFA and LDL-cholesterol than non-carriers [18]. Ordovas et al. reported that male carriers of the –60G allele who were not alcohol drinkers had higher glucose levels than non-carriers [32]. In addition, the C-60G polymorphism is associated with increased waist circumference in lean subjects [33]. The interaction between body fat mass and physical activity is closely associated with the C-60G polymorphism in male carriers. The Quebec Family study showed that men who were G allele carriers were less likely to lose adiposity by physical activity than non-carriers [34]. Talmud et al. found no significant difference in fasting lipid, glucose, BMI, waist/hip ration or blood pressure between C and G allele carriers but the G allele carriers had significant lower HOMA index in healthy young men [35]. Taken together, these previous reports reveal that HSL promoter polymorphisms play a critical role in the regulation of fat and glucose metabolism and are also highly correlated with insulin resistance. The apparent discrepancies between these studies, however, are hard to rationally explain via pathophysiologic mechanisms. To avoid confounding effects, multivariate regression analysis was carried out focusing only on male gender stratified by fasting glucose so insulin resistance is clearly defined. Our results demonstrated different impacts on serum TG by insulin resistance, BMI and the HSL promoter genotype after stratification by serum glucose. Since serum insulin, HOMA-IR and BMI were significantly attributable to a synergistic effect of glucose intolerance and FL, it is necessary to compare the interaction of these confounding factors together on serum TG. We observed no difference in anthropometric or metabolic parameters and related insulin resistance indexes between genotype (CG + GG) and (CC) carriers in the NTG group, except for significantly higher serum TG levels found in carriers of the G allele in the GI group (Additional file 1: Table S1 and S2).

Recent evidence has shown that the accumulation of diacylglycerol in the liver might activate protein kinase C, induce insulin resistance and increase VLDL-TG secretion. HSL is involved in a rate-limiting step to catalyze diacylglycerol and monoacylglycerol into free fatty acids, so HSL activity determines the tissue content of diacylglycerol and the degree of insulin resistance [20,26]. Reid et al. found that hepatic overexpression of HSL promotes β-oxidation of the fatty acid to ameliorate steatosis [36]. Taken together, HSL activity has a critical role in the development of NAFLD. The G allele of the HSL promoter (C-60G) exhibited a 40% decline in promoter activity, which may theoretically cause greater accumulation of diacylglycerol in the liver. This could lead to greater insulin resistance, less β-oxidation and more VLDL-TG secretion. The HSL activity is negatively regulated by insulin and up-regulated by glucose. There are multiple prominent *cis-*regulatory elements, including the insulin response element (IRE), glucose response element (GRE) and fat specific element (FSE), along with other transcriptional factor binding elements, residing in the proximal promoter regions of the human HSL gene. All these *cis-*acting elements act as critical transcriptional regulators for promoter activity [37,38]. A literature review by Ling et al. pointed out that the epigenetic effect of DNA methylation always occurs in CG dinucleotides and is modified by environmental exposure to nutrients in complex multifactorial diseases such as type 2 DM. The relative ratio of insulin, glucose and fat levels may act on the *cis*-regulating elements to modulate promoter function and transcriptional initiation [39]. Our results showed a genetic effect of the HSL promoter (C-60G) regulating serum TG only in the GI group and not in the NGT group. It is rational to speculate that the G allele of the HSL promoter may express a differential susceptibility to the complex interaction between serum insulin, glucose and fat to determine lipolysis, such as lowering lipolytic activity only under a dependable range of insulin resistance. Because this is a cross-sectional study, further prospective and large-scaled study is suggested to verify this preliminary observation.

## Conclusion

The risk interaction between genetic susceptibility, obesity and insulin resistance contribute different impacts on the development of NAFLD. Adipo-IR, rather than HOMA-IR, is proposed as a consistent insulin resistance index for the study of NAFLD. BMI and Adipo-IR were independent risk factors for the formation of FL. In a GI state, HSL promoter (CG + GG) contributed the greatest impact on raising serum TG, followed by FL and Adipo-IR in sequence.

## Abbreviations

NAFLD: Non-alcoholic fatty liver disease; MetS: Metabolic syndrome; NASH: Non-alcoholic steatotic hepatitis; BMI: Body mass index; TG: Triglyceride; FA: Fatty acid; VLDL: Very low-density lipoprotein; FAS: Fatty acid synthase; ATGL: Adipose triglyceride lipase; HSL: Hormone-sensitive lipase; NGT: Normal glucose tolerance; GI: Glucose intolerance; AST: Aspartate aminotransferase; ALT: Alanine aminotransferase; NEFA: Non-esterified fatty acid; HOMA-IR: Homeostatic model assessment of insulin resistance; Adipo-IR: Adipose insulin resistance; FL: Fatty liver; AFL: Absent fatty liver; MONW: Metabolic obese normal weight; IRE: Insulin response element; GRE: Glucose response element; FSE: Fat specific element.

## Competing interests

All of the authors contributed substantially to this study. All of the authors fully declare that there is no conflict of interest prejudicing the text of this article.

## Authors’ contributions

PJH involved research design, quality control of the genetic experiments, and writing the manuscript. ZCC designed the research. WWH searched the literature and analyzed data. YHCY contributed to using analytic tools for statistical analysis and involved the statistical analysis. MYL collected data for analysis. JFH contributed on patients enrollment. KKK supervised the project, edit and revise the manuscript. All authors read and approved the final manuscript.

## Pre-publication history

The pre-publication history for this paper can be accessed here:

http://www.biomedcentral.com/1471-2350/14/54/prepub

## Supplementary Material

Additional file 1: Table S1 Comparison of biochemistry between genotypes (CG+GG) and (CC) of the hormone sensitive lipase (HSL) promoter in the normal glucose tolerance group (n=729). **S2.** Comparison of biochemistry between genotypes (CG+GG) and (CC) of the hormone sensitive lipase (HSL) promoter in the glucose intolerance group (n=299). Click here for file
