# Supplementary material for: Risk interaction of obesity, insulin resistance and hormone-sensitive lipase promoter polymorphisms (LIPE-60 C > G) in the development of fatty liver
Source: BMC Med Genet. 2013 May 20;14:54. doi: 10.1186/1471-2350-14-54 (PMC3673851; doi:10.1186/1471-2350-14-54)
Supplement: Additional file 1: Table S1 — Comparison of biochemistry between genotypes (CG+GG) and (CC) of the hormone sensitive lipase (HSL) promoter in the normal glucose tolerance group (n=729). S2. Comparison of biochemistry between genotypes (CG+GG) and (CC) of the hormone sensitive lipase (HSL) promoter in the glucose intolerance group (n=299). [file 1471-2350-14-54-S1.docx]

**Additional file 1**

**Table S1.**

Comparison of biochemistry between genotypes (CG+GG) and (CC) of the hormone sensitive lipase (HSL) promoter in the normal glucose tolerance group (n=729).

|  | **CC**  **(n=595)** | **CG+GG**  **(n= 134)** | ***P*** |
| --- | --- | --- | --- |
| **Age (yrs)** | 45.3 ± 8.4 | 44.0 ± 8.6 | 0.100 |
| **BMI (kg/m^2^)** | 24.5 ± 3.0 | 24.5 ± 3.3 | 0.823 |
| **Fasting sugar (mg/dl)** | 88.0 ± 7.2 | 87.3 ± 7.2 | 0.317 |
| **Serum insulin (μIU/ml)** | 3.41± 3.83 | 3.17± 3.97 | 0.510 |
| **NEFA (μmol/L)** | 547.1 ± 213.5 | 544.4±225.0 | 0.904 |
| **HOMA-IR** | 0.74 ±0.83 | 0.69 ±0.92 | 0.561 |
| **Adipose-IR** | 1.86 ± 2.14 | 2.22 ± 5.74 | 0.229 |
| **AST (IU/L)** | 17.9 ± 7.2 | 18.5 ± 7.7 | 0.390 |
| **ALT (IU/L)** | 18.9 ± 13.2 | 19.6 ± 12.1 | 0.564 |
| **Cholesterol (mg/dl)** | 180.1 ± 32.5 | 181.5± 30.8 | 0.914 |
| **TG (mg/dl)** | 134.9 ± 99.4 | 132.6 ± 90.9 | 0.800 |
| **HDL (mg/dl)** | 50.8 ± 12.4 | 52.6 ±12.2 | 0.200 |
| **LDL (mg/dl)** | 123.5 ± 33.3 | 124.8 ± 32.9 | 0.724 |
| **Serum creatinine (mg/dl)** | 1.24 ± 0.41 | 1.22 ± 0.13 | 0.627 |

*P* values were obtained from a Student's *t* test for normal distributed variables and a nonparametric Mann–Whitney rank-sum test for non-normally distributed variables

**Table S2.**

Comparison of biochemistry between genotypes (CG+GG) and (CC) of the hormone sensitive lipase (HSL) promoter in the glucose intolerance group (n=299).

|  | **CC**  **(n=236)** | **CG+GG (n=63)** | ***P*** |
| --- | --- | --- | --- |
| **Age (yrs)** | 49.1 ± 8.0 | 49.0 ± 8.7 | 0.923 |
| **BMI (kg/m^2^)** | 24.9 ± 3.5 | 25.8 ± 3.5 | 0.073 |
| **Fasting sugar (mg/dl)** | 125.4 ± 44.6 | 122.8± 35.4 | 0.657 |
| **Serum insulin (μIU/ml)** | 6.31± 10.43 | 7.01± 11.92 | 0.650 |
| **NEFA (μmol/L)** | 620.2 ± 251.9 | 657.7±264.2 | 0.311 |
| **HOMA-IR** | 2.01 ±3.61 | 2.36 ±5.50 | 0.553 |
| **Adipose-IR** | 3.63 ± 4.96 | 4.05 ± 4.98 | 0.567 |
| **AST (IU/L)** | 20.1 ± 11.2 | 20.8 ± 11.9 | 0.672 |
| **ALT (IU/L)** | 22.5 ± 19.7 | 22.2 ± 16.2 | 0.939 |
| **Cholesterol (mg/dl)** | 181.8 ± 33.0 | 189.9± 34.6 | 0.098 |
| **TG (mg/dl)** | 142.6 ± 82.1 | 176.5 ± 108.44 | **0.023** |
| **HDL (mg/dl)** | 49.6 ± 10.8 | 46.1 ± 9.6 | 0.087 |
| **LDL (mg/dl)** | 124.0 ± 31.3 | 128.6 ± 35.6 | 0.453 |
| **Serum creatinine (mg/dl)** | 1.25 ± 0.18 | 1.29 ± 0.16 | 0.139 |

*P* values were obtained from a Student's *t* test for normal distributed variables and a nonparametric Mann–Whitney rank-sum test for non-normally distributed variables
